# Performance-based robotic assistance during rhythmic arm exercises

**DOI:** 10.1186/s12984-016-0189-7

**Published:** 2016-09-13

**Authors:** Patricia Leconte, Renaud Ronsse

**Affiliations:** 1 Université catholique de Louvain, Center for Research in Mechatronics, Institute of Mechanics, Materials and Civil Engineering, Place du Levant 2, Louvain-la-Neuve, 1348 Belgium; 2 Université catholique de Louvain, Institute of Neuroscience, Tour Pasteur - Avenue Mounier 53, Brussels, 1200 Belgium; 3 Université catholique de Louvain, Louvain Bionics, Place du Levant 2, Louvain-la-Neuve, 1348 Belgium

**Keywords:** Neurorehabilitation, Assist-as-needed, Performance-based assistance, Robotics

## Abstract

**Background:**

Rhythmic and discrete upper-limb movements are two fundamental motor primitives controlled by different neural pathways, at least partially. After stroke, both primitives can be impaired. Both conventional and robot-assisted therapies mainly train discrete functional movements like reaching and grasping. However, if the movements form two distinct neural and functional primitives, both should be trained to recover the complete motor repertoire. Recent studies show that rhythmic movements tend to be less impaired than discrete ones, so combining both movement types in therapy could support the execution of movements with a higher degree of impairment by movements that are performed more stably.

**Methods:**

A new performance-based assistance method was developed to train rhythmic movements with a rehabilitation robot. The algorithm uses the assist-as-needed paradigm by independently assessing and assisting movement features of smoothness, velocity, and amplitude. The method relies on different building blocks: (i) an adaptive oscillator captures the main movement harmonic in state variables, (ii) custom metrics measure the movement performance regarding the three features, and (iii) adaptive forces assist the patient. The patient is encouraged to improve performance regarding these three features with assistance forces computed in parallel to each other. The method was tested with simulated jerky signals and a pilot experiment with two stroke patients, who were instructed to make circular movements with an end-effector robot with assistance during half of the trials.

**Results:**

Simulation data reveal sensitivity of the metrics for assessing the features while limiting interference between them. The assistance’s effectiveness with stroke patients is established since it (i) adapts to the patient’s real-time performance, (ii) improves patient motor performance, and (iii) does not lead the patient to slack. The smoothness assistance was by far the most used by both patients, while it provided no active mechanical work to the patient on average.

**Conclusion:**

Our performance-based assistance method for training rhythmic movements is a viable candidate to complement robot-assisted upper-limb therapies for training a larger motor repertoire.

## Background

Rhythmic and discrete movements have recently been recognized as two of the most fundamental units of the upper- [1] and lower-limb [2] motor repertoire. Rhythmic movements capture periodic movements like hammering or scratching, while discrete movements capture movements between a succession of postures with zero velocity and acceleration, like reaching and pointing [3, 4]. These two fundamental motor primitives are controlled by distinct neural circuitries, at least partially [3, 5–14]. For example, previous research with healthy subjects showed that (i) discrete movements require more cortical activity than rhythmic ones [7], and (ii) no learning transfer occurs from rhythmic to discrete movements and only a little transfer occurs from discrete to rhythmic movements when they are executed in altered visual or haptic conditions [13, 14].

After a stroke, both rhythmic and discrete movements can be impaired [15–23]. Recently, we compared the performance in executing both movements in the same stroke population. As a main conclusion, we found that rhythmic arm movements are less affected than discrete ones. In particular, stroke preserved the smoothness of rhythmic movements so that fewer submovements were identified than in the discrete counterparts [24]. However, rhythmic movements were impaired compared to healthy subjects. Stroke patients decelerated more than healthy subjects at the movement reversal, and some patients displayed a larger amount of submovements.

If rhythmic and discrete movements are two distinct primitives, they deserve specific and differentiated training to permit the full recovery of the complete motor repertoire. This is a necessary condition to recover autonomy life activities requiring a combination of rhythmic and discrete movements (such as wiping a table or playing the piano [5]).

Most post-stroke therapies tend to focus on functional and thus mainly discrete movements [25–27], although some previous contributions did focus on upper-limb rhythmic movement training. Interestingly, they all tended to display an improvement in motor skills [28–34]. For instance, [28, 29] highlighted that the intensity of the training is critical to enhance motor skills. In [33], the authors compared bilateral arm training with auditory cueing (BATRAC) to dose-matched therapeutic exercises and concluded that none was superior to the other, although the adaptations in brain activation were greater after BATRAC. Whether this result is due to the rhythmic nature of the movement, its bimanual nature, the auditory cueing, or a combination of these features, is however difficult to establish, since these are closely intertwined in BATRAC.

The current state-of-art of rhythmic upper-limb movement therapy calls thus for the development of post-stroke therapies tailored to unilateral rhythmic movement training, in order to study their exact effect on motor skills. The development of such a therapy is presented in this paper.

Robotic devices are particularly suited for implementing post-stroke therapies, with a specific focus on movement intensity. Rehabilitation robots enable patients to practice well-specified motor actions and can deliver an appropriate amount of assistance to help patients in improving their motor behavior [17, 35–42]. Motor performance can be computed in real-time by the robot controller, allowing for continuous adaptation of the type and amount of assistance. The patient only receives the necessary support and is prevented from slacking [36, 43]. In the literature, this is often referred to as the “slacking hypothesis,” which suggests that too much assistance will cause a progressive decrease in patient effort to accomplish a desired task and reduce motor recovery. This assistance principle is also called “assistance as needed” and has progressively emerged as a hallmark of successful robot-assisted therapies [35, 36, 43, 44]. This principle lies also at the core of the present contribution.

Most upper-limb robot-assisted therapies are designed for discrete movement training and implement the assist-as-needed principle through different strategies. One type of strategy delivers assistance proportional to the trajectory error with respect to a predefined trajectory [42, 45–50]. Another assistance approach relies on dynamical systems and adapts the assistance parameters as a function of the patient performance [45]. Other approaches tune the amount of assistance across sessions as a function of the performance during the preceding session [45, 51]. Another method [47] performs an online adaptation of the amount of support depending on the activity (for a survey, see [35]).

In contrast with these approaches, a rhythmic movement therapy should exploit the cyclic nature of the movement to anticipate the future trajectory based on previous cycles. This can be achieved by using adaptive oscillators [52]. These mathematical tools are particularly suited to track the main features of a typical rhythmic movement (like amplitude and frequency). This continuous assessment allows the robot to constantly seek to improve movement features with the appropriate amount and type of assistance. Moreover, this approach naturally allows for trajectory-free assistance algorithms so that the therapist does not have to specify an arbitrary target trajectory for the patient to follow. The patient receives assistance to improve the impacted movement features, but is left free to produce any rhythmic trajectory.

Our previous work already paved the way in using adaptive oscillators to deliver trajectory-free assistance for upper- [53] and lower-limb [54, 55] rhythmic movements. These contributions focused on movement assistance for healthy subjects, showing evidence of decreases in metabolic consumption when the assistance was switched on. The present study is the first to propose a metric-based assistance method for patients with motor disorders, with emphasis on the potential to assist different rhythmic movement features as a function of the patient needs.

This paper outlines the performance-based assistance method and its mathematical foundations in details. The method was validated with data from simulations and a pilot study with two stroke patients with upper-limb impairments is also reported. The proposed performance-based assistance method can (i) enhance motor-performance, (ii) give appropriate assistance according to patient performance, and (iii) maintain active patient participation in the task so that no slacking effect occurs.

## Methods

**Fig. 1:**
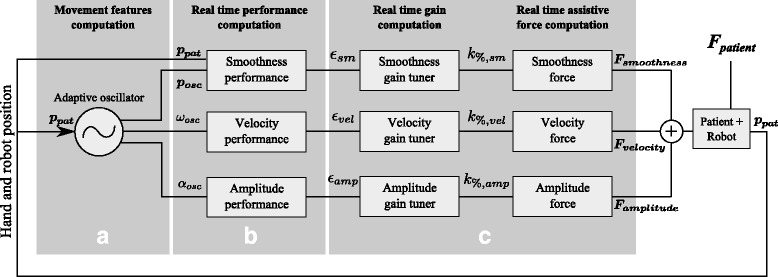
Methodology. Outline of the overall control strategy of the performance-based assistance. First, the movement features are computed by the adaptive oscillator (**a**) and serve as input to compute the real-time performance in smoothness, velocity, and amplitude (**b**). These features are then used to compute the gains to tune the level of the assistance forces in smoothness, velocity, and amplitude (**c**). These three forces are eventually summed up and delivered to the patient

The main interest of the developed method is that it can independently assist different movement features, only if needed. In particular, the method implements parallel strategies to assist the patient in improving performance regarding movement smoothness, velocity, and amplitude. Therefore, the method requires measuring (Fig. 1a) and quantifying (Fig. 1b) the amplitude, velocity, and smoothness features of patient movement in real-time in order to assess the corresponding performance. The method must also compute and deliver the appropriate amount of assistance in amplitude, velocity, and/or smoothness as a function of the performance (Fig. 1c).

The most comprehensible way to develop the proposed assistance method requires focusing on a simple rhythmic movement. This movement is a rhythmic circular trajectory executed with the upper-limb. It advantageously combines different desirable features: it is harmonic by essence, involves no contact with the environment, and requires the coordination of several degrees of freedom. The parameters shaping this movement are the amplitude, offset (i.e. position of the circle center), and rotational frequency. The desired movement kinematics are characterized by the vectorial position and velocity in Cartesian space, as a function of time:

1 $$\begin{array}{*{20}l} \boldsymbol{p}_{\boldsymbol{d}} &= \left(\begin{array}{c} x_{d} \\ y_{d} \end{array}\right) = \left(\begin{array}{c} \varphi_{x,d} +\alpha_{d} \sin{\omega_{d} t}\\ \varphi_{y,d} -\alpha_{d} \cos{\omega_{d} t} \end{array}\right)  \end{array} $$

2 $$\begin{array}{*{20}l} \boldsymbol{\dot{p}}_{\boldsymbol{d}} &= \left(\begin{array}{c} \dot{x}_{d} \\ \dot{y}_{d} \end{array}\right) = \left(\begin{array}{c} \alpha_{d} \omega_{d} \cos{\omega_{d} t}\\ \alpha_{d} \omega_{d} \sin{\omega_{d} t} \end{array}\right)  \end{array} $$

where *α*_*d*_ is the movement amplitude, *ω*_*d*_ is the angular frequency and *φ*_*x,d*_ and *φ*_*y*,*d*_ are the offsets along the *x*- and *y*-axes, respectively.

### Real-time measurement of the movement features

Adaptive oscillators are particularly suited for estimating the real-time movement parameters of a rhythmic movement [52, 56–58]. From the ideal movement in (1), an adaptive oscillator can learn an equivalent movement captured by the following model:

3 $$\begin{array}{@{}rcl@{}} \boldsymbol{p}_{\boldsymbol{osc}} = \left(\begin{array}{c} x_{osc} \\ y_{osc} \end{array} \right) = \left(\begin{array}{c} \varphi_{x,osc} +\alpha_{osc} \sin{\phi_{osc}}\\ \varphi_{y,osc} -\alpha_{osc} \cos{\phi_{osc}} \end{array}\right) \end{array} $$

where *α*_*osc*_ is the learned amplitude, *ϕ*_*osc*_ is the learned phase (the time integral of the learned angular velocity *ω*_*osc*_), and *φ*_*x*,*osc*_ and *φ*_*y*,*osc*_ are the learned offsets.

These variables are the state variables of the adaptive oscillator. Their learning dynamics are governed by:

4 $$\begin{array}{@{}rcl@{}} \dot{\phi}_{osc} &=& \omega_{osc} + \nu_{\phi} \left(F_{x}\frac{\cos{\phi_{osc}}}{\alpha_{osc}} + F_{y}\frac{\sin{\phi_{osc}}}{\alpha_{osc}}\right)  \end{array} $$

5 $$\begin{array}{@{}rcl@{}} \dot{\omega}_{osc} &=& \nu_{\omega} \left(F_{x}\frac{\cos{\phi_{osc}}}{\alpha_{osc}}+ F_{y} \frac{\sin{\phi_{osc}}} {\alpha_{osc}}\right)  \end{array} $$

6 $$\begin{array}{@{}rcl@{}} \dot{\alpha}_{osc} &=& \eta_{\varphi} \left(F_{x}\sin{\phi_{osc}} - F_{y} \cos{\phi_{osc}}\right) \end{array} $$

7 $$\begin{array}{@{}rcl@{}} \dot{\varphi}_{osc,x} &=& \eta_{\varphi} F_{x} \end{array} $$

8 $$\begin{array}{@{}rcl@{}} \dot{\varphi}_{osc,y} &=& \eta_{\varphi} F_{y}  \end{array} $$

where *F*_*x*_=*x*−*x*_*osc*_ and *F*_*y*_=*y*−*y*_*osc*_ are the error signals that capture the difference between the oscillator input (*x* and *y*, i.e. either the simulated or real 2D hand position in the experiments reported in this paper) and the estimated position output.

By properly tuning the learning gains *ν*_*ϕ*_, *ν*_*ω*_, and *η*_*φ*_, the output signal will synchronize with the input signal after only a few cycles while learning the actual input features in the internal state variables. The learning dynamics can be finely tuned by adapting the learning gains [56]. If the input signal contains higher harmonics in its frequency spectrum, the oscillator output can be considered as a “smoothed” or filtered version of the input, although both will be phase-synched on average. In sum, this adaptive oscillator provides real-time estimates of the parameters of a time-varying periodic signal while filtering this signal in its output.

The method can also provide a smooth estimate of the signal derivatives [56]. For instance, the estimate of the signal’s first derivative is obtained by analytical time-differentiation of the augmented phase oscillator (3):

9 $$\begin{array}{@{}rcl@{}} \boldsymbol{\dot{p}}_{\boldsymbol{osc}} = \left(\begin{array}{c} \dot{x}_{osc} \\ \dot{y}_{osc} \end{array} \right) = \left(\begin{array}{c} \alpha_{osc}\omega_{osc} \cos{\phi_{osc}}\\ \alpha_{osc}\omega_{osc} \sin{\phi_{osc}} \end{array} \right)  \end{array} $$

More details about the mathematical foundations of these adaptive oscillators can be found elsewhere [52, 55].

The adaptive oscillator is used for two different purposes throughout the proposed therapy: for measuring the initial abilities of the patient through an initialization phase and for measuring the real-time performance (smoothness, velocity, and amplitude) of the patient during the robot-assisted rhythmic exercises (Fig. 1a). The second item is further detailed in the next section.

### Real-time performance computation

#### Real-time smoothness metric

Assessing the smoothness of a discrete movement after completion (off-line) has been the topic of many former studies. The challenge lies in determining a dimensionless metric that is independent of the movement amplitude and duration while having a univocal response to the motion characteristics: the smoothness has to monotonically decrease when the amount of submovements and the inter-submovement duration decrease [59, 60]. Several metrics were proposed and analyzed to quantify the movement smoothness after the completion of the whole movement, such as the normalized mean absolute jerk, number of peaks in the velocity profile, logarithmic dimensionless jerk, spectral arc length, and speed arc length [18, 20, 59, 61].

Real-time measurement of rhythmic movement smoothness is required to continuously adapt the assistance. The metrics mentioned above are thus ineligible since they can only provide a smoothness estimate after the completion of one or several movement cycles. Hence, we propose measuring the smoothness performance in continuous-time by comparing the actual velocity of the patient, $\dot {x}_{pat}$ and $\dot {y}_{pat}$, with the desired velocity computed by the adaptive oscillator (9); i.e., the filtered version of the patient actual velocity. This error is normalized with respect to the angular velocity (the product of *ω*_*osc*_ and *α*_*osc*_) to obtain a dimensionless metric:

10 $$\begin{array}{@{}rcl@{}} \eta &=& \frac{\sqrt{\left(\dot{x}_{osc} - \dot{x}_{pat}\right)^{2} + \left(\dot{y}_{osc} - \dot{y}_{pat}\right)^{2}}}{\omega_{osc}\alpha_{osc}} \end{array} $$

Next, we compute the numerical differentiation of this velocity error signal and normalize it again with the angular velocity:

11 $$\begin{array}{@{}rcl@{}} \xi &=& \frac{d\eta}{dt} \frac{1}{\omega_{osc}} \end{array} $$

Consequently, if a patient smoothly accelerates or decelerates, this dimensionless differentiated velocity error *ξ* will stay bounded. Finally, the absolute value of *ξ* is low-pass filtered to obtain a quasi-real-time smoothness measurement. The time constant (and bandwidth) of this filter is proportional to *ω*_*osc*_, which normalizes the filter response with respect to the movement frequency:

12 $$\begin{array}{@{}rcl@{}} \dot{\epsilon}_{sm} &=& a \omega_{osc} (|\xi| - \epsilon_{sm})  \end{array} $$

In (12), *a* is a constant gain that tunes the frequency-dependent time constant of the filter. If the movement is performed as an ideal circle of constant tangential velocity, *η*, *ξ*, and thus *ε*_*sm*_ will converge towards 0. Moreover, a circular movement performed with a constant acceleration will cause *ε*_*sm*_ to converge towards 0 since the velocity error *η* will reach a plateau. The more fluctuations there are in the velocity error, the higher *ε*_*sm*_ is.

#### Real-time velocity error

The real-time velocity error is simply obtained from the difference between the desired movement frequency *ω*_*d*_ and the actual one estimated by the adaptive oscillator:

13 $$\begin{array}{@{}rcl@{}} \epsilon_{vel} = \omega_{d}- \omega_{osc}  \end{array} $$

#### Real-time amplitude error

Similarly to the real-time velocity error, the real-time amplitude error is the difference between the estimated amplitude of the circles drawn by the patient, *α*_*osc*_, and the desired amplitude, *α*_*d*_:

14 $$\begin{array}{@{}rcl@{}} \epsilon_{amp} = \alpha_{d}- \alpha_{osc}  \end{array} $$

### Performance-based assistance forces

The estimates of different movement features allow for three types of assist-as-needed forces to be delivered to the patient. “Smoothness assistance” is delivered if the performed movements are detected to be jerky and deviate too much from the ideal movement given by (1). “Velocity assistance” is delivered if the performed movements are too slow, which is determined by the detected movement tangential velocity (the product of the movement frequency *ω*_*osc*_ and amplitude *α*_*osc*_) being too far below the desired one *α*_*d*_*ω*_*d*_. “Amplitude assistance” is delivered if the performed movements are too small, i.e. if the detected movement amplitude *α*_*osc*_ is too far below the desired one *α*_*d*_. Since the types of assistance are managed by parallel algorithms, they can occur simultaneously (Fig. 1c):

15 $$\begin{array}{@{}rcl@{}} \boldsymbol{F}_{\boldsymbol{robot}} = \boldsymbol{F}_{\boldsymbol{smoothness}} + \boldsymbol{F}_{\boldsymbol{velocity}} + \boldsymbol{F}_{\boldsymbol{amplitude}}  \end{array} $$

where ***F***_***robot***_ is the total assistance force being delivered by the robot.

#### Smoothness assistance

**Fig. 2:**
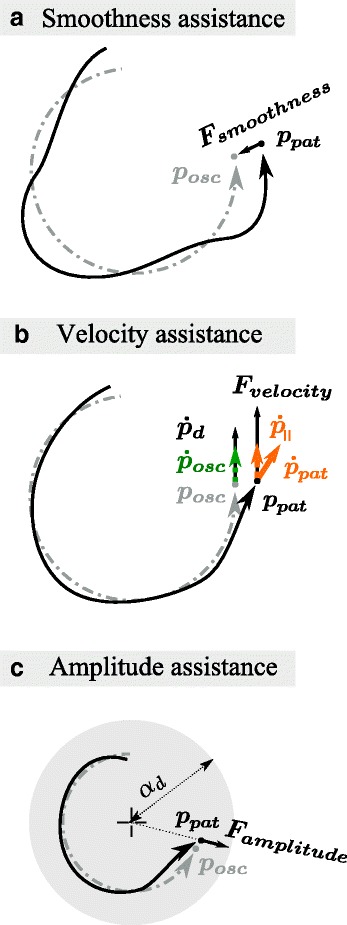
Assistance forces. The three assistance forces that can be provided to the patient to enhance the movement features are presented in the three panels. **a** The smoothness assistance attracts the patient to the smooth position produced by the adaptive oscillator, **b** the velocity assistance tangentially supports the patient to reach the desired velocity, and **c** the amplitude assistance is a radial force towards the desired amplitude

Smoothness assistance is provided by a virtual damped spring between the current estimated position provided by the adaptive oscillator and the actual position of the patient (Fig. 2a). This type of assistance guides the patient hand to a “smoothed version” of its own movement since the adaptive oscillator synchronizes to the input while filtering out the frequency content from its main harmonic. The oscillator neither lags behind nor leads the patient hand on average since it is phase-synched to it. Accordingly, the force applied to the patient hand (***F***_***smoothness***_) is equal to:

16 $$\begin{array}{@{}rcl@{}} \boldsymbol{F}_{\boldsymbol{smoothness}} = k_{sm}\left(\boldsymbol{p}_{\boldsymbol{osc}} - \boldsymbol{p}_{\boldsymbol{pat}}\right) + c \left(\boldsymbol{\dot{p}}_{\boldsymbol{osc}} - \boldsymbol{\dot{p}}_{\boldsymbol{pat}}\right)  \end{array} $$

where *k*_*sm*_ is the adaptive stiffness of the spring and *c* is the damping coefficient. To render the behavior of a spring with critical damping, $c = 2\sqrt {m k_{sm}}$ (where *m* is the equivalent virtual mass of the robot, see Section ’Validation with experimental data’). In (16), ***p***_***pat***_ and $\boldsymbol {\dot {p}}_{\boldsymbol {pat}}$ denote the measured vectorial position and velocity of the patient hand, respectively.

Due to the adaptive nature of the oscillator used to compute the movement features, ***p***_***osc***_ and $\boldsymbol {\dot {p}}_{\boldsymbol {osc}}$ constantly adapt to the movement being performed by the patient. Therefore, the smoothness assistance should equally accelerate and decelerate the patient hand during steady-state behavior. Said differently, it should provide no positive mechanical work to the patient on average. In sum, the smoothness assistance helps in making a smoother movement but does not directly make it larger or faster.

#### Velocity assistance

If the patient is not able to perform the desired movement at the desired frequency, velocity assistance can be provided. This assistance is implemented through a force that is tangential to the desired velocity profile:

17 $$\begin{array}{@{}rcl@{}} \boldsymbol{F}_{\boldsymbol{velocity}} &=& k_{vel} (\boldsymbol{\dot{p}}_{\boldsymbol{d\parallel}}-\boldsymbol{\dot{p}}_{\boldsymbol{\parallel}})  \end{array} $$

if $|\boldsymbol {\dot {p}}_{\boldsymbol {d\parallel }}|>|\boldsymbol {\dot {p}}_{\boldsymbol {\parallel }}|$; otherwise, ***F***_***velocity***_=0. *k*_*vel*_ denotes the adaptive gain (Fig. 2b), $\boldsymbol {\dot {p}}_{\boldsymbol {\parallel }}$ is the orthogonal projection of $\boldsymbol {\dot {p}}_{\boldsymbol {pat}}$ on $\boldsymbol {\dot {p}}_{\boldsymbol {d\parallel }}$, and $\boldsymbol {\dot {p}}_{\boldsymbol {d\parallel }}$ is the desired velocity vector, which is tangential to the ideal circle being learned by the adaptive oscillator:

18 $$\begin{array}{@{}rcl@{}} \boldsymbol{\dot{p}}_{\boldsymbol{\parallel}} &=& (\boldsymbol{\dot{p}}_{\boldsymbol{pat}} \cdot \boldsymbol{e}_{\boldsymbol{\parallel}})\cdot \boldsymbol{e}_{\boldsymbol{\parallel}} \end{array} $$

19 $$\begin{array}{@{}rcl@{}} \boldsymbol{\dot{p}}_{\boldsymbol{d\parallel}} &=& v_{d} \boldsymbol{e}_{\boldsymbol{\parallel}} \end{array} $$

where *v*_*d*_=*ω*_*d*_*α*_*d*_ and

20 $$\begin{array}{@{}rcl@{}} \boldsymbol{e}_{\boldsymbol{\parallel}} &=& \frac{\boldsymbol{\dot{p}}_{\boldsymbol{osc}}}{\parallel\boldsymbol{\dot{p}}_{\boldsymbol{osc}}\parallel}. \end{array} $$

In sum, this assistance provides a smooth longitudinal force if the patient’s tangential velocity is below a desired threshold.

#### Amplitude assistance

Finally, if the amplitude of the patient movement (captured by the adaptive oscillator state variable *α*_*osc*_) is below the desired amplitude, a radial force is provided by the robot to encourage larger movements. Therefore, if the actual position of the patient is inside the minimal amplitude circle (|***α***_***pat***_|<|***α***_***d⊥***_|), assistance is delivered through a force:

21 $$\begin{array}{@{}rcl@{}} \boldsymbol{F}_{\boldsymbol{amplitude}} = k_{amp} (\boldsymbol{\alpha}_{\boldsymbol{d\perp}} - \boldsymbol{\alpha}_{\boldsymbol{pat}})  \end{array} $$

Otherwise, ***F***_***amplitude***_=0. In (21), ***α***_***pat***_ is the vector between the center of the circle ***φ***_***osc***_=(*φ*_*x*,*osc*_,*φ*_*y*,*osc*_) and the actual position of the patient, ***α***_***d⊥***_ is the vector from the center of the circle towards the actual position of the adaptive oscillator (with length *α*_*d*_), and *k*_*amp*_ is the adaptive gain (Fig. 2c). In mathematical terms,

22 $$\begin{array}{@{}rcl@{}} \boldsymbol{\alpha}_{\boldsymbol{d\perp}} &=& \alpha_{d} \boldsymbol{e}_{\boldsymbol{\perp}}, \end{array} $$

23 $$\begin{array}{@{}rcl@{}} \boldsymbol{\alpha}_{\boldsymbol{pat}} &=& (\boldsymbol{p}_{\boldsymbol{pat}}-\boldsymbol{\varphi}_{\boldsymbol{osc}}) \end{array} $$

and

24 $$\begin{array}{@{}rcl@{}} \boldsymbol{e}_{\boldsymbol{\perp}} = \frac{\boldsymbol{p}_{\boldsymbol{pat}} - \boldsymbol{\varphi}_{\boldsymbol{osc}}} {\parallel\boldsymbol{p}_{\boldsymbol{pat}} - \boldsymbol{\varphi}_{\boldsymbol{osc}}\parallel}. \end{array} $$

#### Gain tuners

**Fig. 3:**
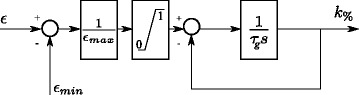
Performance-based gain tuners. Low-pass filters tune the amount of assistance *k*_*%*_ delivered to the patient according to the corresponding performance *ε* with a time constant of *τ*_*g*_ seconds

To prevent the patients from slacking [36] and to mimic the way a physical therapist would help a patient during an exercise, the levels of assistance have to evolve as a function of the real-time patient performance. If a performance metric is low, the gain of the corresponding assistance has to increase. If the performance improves, the gain should decrease in order to encourage the patient to stay active. Three parallel low-pass filters are thus used to tune the three gains of the assistance forces: *k*_*sm*_ (16), *k*_*vel*_ (17), and *k*_*amp*_ (21); see Fig. 3. They dynamically evolve between 0 (no assistance if the performance is good) and a maximum value (if the performance is poor) through a scaling factor *k*_*%*,*metric*_∈ [0,1], such that *k*_*metric*_=*k*_*%*,*metric*_*k*_*max*,*metric*_. The input of these three filters are the corresponding *ε*_*metric*_: the performance indices *ε*_*sm*_ (12), *ε*_*vel*_ (13), and *ε*_*amp*_ (14). *ε*_*min*,*metric*_ is the minimum error tolerated before *k*_*%*,*metric*_ increases, and *ε*_*max*,*metric*_ is the maximal error that causes *k*_*%*,*metric*_ to saturate to 1. The time constant of these filters is set to *τ*_*g*_ seconds.

### Validation with simulated data

Before validating the performance-based assistance with patient data, the adaptive oscillator, smoothness metric, and performance-based assistance were validated with simulated jerky circular movements. These unideal rhythmic signals were generated by augmenting a smooth rhythmic circular movement (similar to (1)) with lateral and longitudinal errors of time-varying amplitude and frequency captured by:

25 $$\begin{array}{@{}rcl@{}} \alpha_{jerky} &=&\bar{\alpha} \left(1+\delta_{lat}\sin{(\omega_{lat} t)}\right), \end{array} $$

26 $$\begin{array}{@{}rcl@{}} \omega_{jerky} &=& \bar{\omega} \left(1+\delta_{long}\cos{\left(\omega_{long}t \right)}\right), \end{array} $$

27 $$\begin{array}{@{}rcl@{}} \phi_{jerky}&=& \int{\omega_{jerky}} \mathrm{d}t \end{array} $$

$\bar {\alpha } $ and $\bar {\omega }$ are the mean amplitude and frequency of the simulated jerky movement, while *ω*_*lat*_ and *ω*_*long*_ capture the frequency of the lateral errors and longitudinal errors, and *δ*_*lat*_ and *δ*_*long*_ capture the amplitude of the lateral and longitudinal errors, respectively (Fig. 6, top).

The longitudinal velocity *v*_*long*_ of the simulated jerky movement with longitudinal errors is equal to:

28 $$\begin{array}{@{}rcl@{}} v_{long} &=& \bar{\alpha} \bar{\omega} \left[1+\delta_{long} \cos{\left(\omega_{long} t\right)}\right] \end{array} $$

which is obtained by time-differentiating (1) with *ϕ*_*d*_=*ϕ*_*jerky*_ and *α*_*d*_=*α*_*jerky*_. By varying *δ*_*long*_, the longitudinal velocity error varies proportionally to the mean longitudinal velocity $\bar {\alpha }\bar {\omega }$. These simulated jerky signals were first used to validate the convergence of the adaptive oscillator towards the average amplitude $\bar {\alpha }$ and frequency $\bar {\omega }$. The values of the parameters that were used during the learning phase are shown in Table 1 (“Sim. data and learning” column). $\bar {\alpha }$ was set to 5 cm and $\bar {\omega }$ to 3.14 rad/s.

**Table 1:** List of parameters used to compute the smoothness metric (*ε*_*sm*_), output of the adaptive oscillator (AO), and gains of the assistance forces (gain tuner)

	Parameter	Sim. data and learning	Pat. data
*ε*_*sm*_	*a*	0.004	0.004
AO	*ν*_*ϕ*_	7.33	2.2
	*ν*_*ω*_	2.22	0.2
	*η*	0.67	0.2
Gain tuner	*τ*_*g*_	1	1
	*ε*_*min*,*sm*_	0.0025	0.0025
	*ε*_*min*,*vel*_	0	0
	*ε*_*min*,*amp*_	0	0
	*ε*_*max*,*sm*_	0.009	0.009
	*ε*_*max*,,*vel*_	1	1
	*ε*_*max*,*amp*_	1	1

The column “Sim. data and learning” contains the values of the parameters used in the validation with simulated data and in the learning phase of the patient performance. The second column “Pat. data” shows values used during the assisted trials with the pilot patients. The adaptive oscillator gains were tuned using a published method [56] to obtain fast (*τ*=3s) and slow (*τ*=10s) learning time constants, respectively for both columns

Next, in order to validate the smoothness metric, a variety of simulated movements were computed with different magnitudes of lateral and longitudinal errors. Ideally, the metric should increase when the amplitude (*δ*_*lat*_) or frequency (*ω*_*lat*_) of the lateral error increase, or when *δ*_*long*_ or *ω*_*long*_ increase for the longitudinal error. Simulations were achieved by testing different sets of parameters (*δ*_*lat*_∈[ 0,3.75] cm, $\omega _{lat}\in \, [0,4]\bar {\omega }$, *δ*_*long*_∈ [ 0,3.75], and $\omega _{long}\in [\!0,3]\bar {\omega }$), as well as for different movement amplitudes ($\bar {\alpha }\in \, [4,10]$ cm) and frequencies ($\bar {\omega }\in \, [\pi /2,3\pi ]$ rad/s). The other parameters are displayed in Table 1 (“Sim. data and learning” column).

Finally, to validate the performance-based assistance, we built a signal composed of different types of errors. This signal was composed of (i) five cycles of unperturbed circular rhythmic movement ($\bar {\alpha } = 5$ cm and $\bar {\omega } = 3.14$ rad/s, *δ*_*lat*_=0 and *δ*_*long*_=0), followed by (ii) five cycles of smaller circular rhythmic movements ($\bar {\alpha } =3$ cm), (iii) five cycles of slow movements ($\bar {\omega }=2.09$), (iv) five cycles of jerky movements with lateral errors (*δ*_*lat*_=0.2, $\omega _{lat}=4\bar {\omega }$, $\bar {\alpha } = 5$ cm, $\bar {\omega } = 3.14 $), and (v) five cycles of jerky movements with longitudinal errors (*δ*_*long*_=0.6, $\omega _{long}=4\bar {\omega }$, $\bar {\alpha } = 5$ cm and $\bar {\omega } = \pi $ rad/s). These simulated pathological movements were separated from each other by five periods of unperturbed rhythmic circular movements.

This signal was used to compute the real-time performance metrics and evolution of the corresponding gains of the performance-based assistances. The minimum desired amplitude *α*_*d*_ was set to 4 cm, and the minimum desired frequency *ω*_*d*_ was 2.8. The values of the parameters used to compute the metrics and gains are displayed in Table 1 (“Sim. data and learning” column). In all simulations, no assistive force was actually delivered since the kinematics was prescribed to follow the simulated signals.

### Validation with experimental data

Finally, the performance-based assistance was implemented on an end-effector robot, and the protocol was tested with two stroke patients (see Table 2). Both patients gave written informed consent to participate in the study, which was approved by the scientific and ethical committees of the Université catholique de Louvain.

**Table 2:** Patients characteristics

	Age	Gender	Time post-stroke	FMA	Hemiplegic side
P1	61	Male	4y	23	Left
P2	50	Male	1y	24	Right

#### Experimental apparatus

**Fig. 4:**
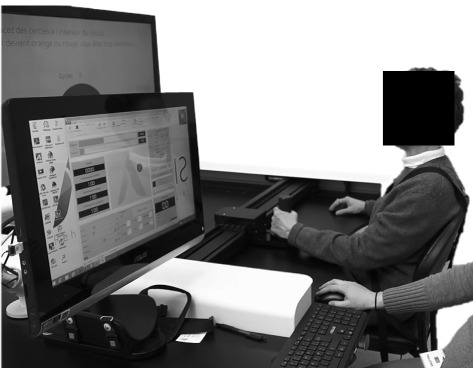
Experimental apparatus. Planar end-effector robot (REAplan) used to validate the performance-based assistance. The patient is seated in front of the device and holds the handle with the paretic hand

The robotic assistance was implemented on an upper-limb end-effector robot, REAplan, which was developed within our university. This device was designed to quantify upper limb impairments of disabled patients [22, 62] and to provide robot-assisted therapies to the same populations [21]. The robot is composed of (i) a height-adjustable horizontal table, (ii) a handle equipped with strain gauges force sensors held by the participant, (iii) two MAXON motors (EC40 170W 42VDC with planetary gearheads GP42C6/1 and encoder HEDL5560 1000imp 2ch) actuating the handle in the horizontal plane, (iv) a flat screen and loudspeakers in front of the participant, which can provide visual feedback of the position of the handle and any other visual or auditory information, and (v) an interface next to the robot for the therapist. All these components are shown in Fig. 4.

When controlled to be transparent, i.e. to deliver no force hindering the patient’s voluntary movements, the actual robot impedance was identified to be similar to the one of a damped mass. The residual force **F**_**res**_ thus obeyed a dynamical equation like $\mathbf {F}_{\mathbf {res}} = m \ddot {\mathbf {p}}_{\mathbf {pat}} + b \dot {\mathbf {p}}_{\mathbf {pat}}$. The residual virtual mass of robot was identified to be equal to *m*=0.45 kg and the virtual damping factor to *b*=2.26 kg/s.

Patients were too weak to hold the handle by themselves with their paretic hand. Consequently, it was strapped to the handle, and their forearm was supported by a plate rigidly attached to the handle.

#### Proposed therapy

After seating the patient in front of the robot, his initial abilities were computed. Patients were asked to perform ten rhythmic circular movements at a comfortable speed and amplitude while moving the robot handle with their paretic hand. The robot was configured to be transparent in this case. During this initialization phase, the adaptive oscillator learned the movement features of the patient with dynamics governed by Eq. (3) (see “Sim. data and learning” column in Table 1 for the numerical values of the parameters). For the next phase, the therapy itself, the desired amplitude *α*_*d*_ and tangential velocity *v*_*d*_=*ω*_*d*_*α*_*d*_ were set 10 % higher than those obtained after this initialization. This increase was administered in order to make the patient performing close to their maximum capacity.

Next, patients were asked to perform rhythmic circular movements while receiving assistance with the desired movement parameters during half of the trials. The screen displayed a circular path with inner radius set at *α*_*d*_. The width of this path was set to 4 *cm*. Patients were asked to perform circular movements while staying inside the path. The path was displayed in red if they moved too slowly on average (*ω*_*osc*_<*ω*_*d*_), while the path was green if they were going faster than *ω*_*d*_. The patients were instructed to maintain the green color as much as possible.

This task was continuously performed by the patients during 40 seconds and was repeated ten times, i.e. 10 trials of 40 seconds. They received assistance during half of these trials with random distribution in order to prevent them to display different behaviors in anticipation of a trial with or without assistance. They received at least one minute of rest between two consecutive trials.

The therapist interface served as control unit, i.e. to select if the assistance has to be delivered or not, and to follow the real-time performances of the patient. Importantly, the therapist was instructed to not to mention to the patient if he was receiving assistance or not, and to deliver no other feedback than what was displayed on his screen.

To validate our approach, we show that the assistance (i) adapts to the real-time motor performance, (ii) enhances the global motor performance, (iii) does not increase with time (no slacking), and that (iv) the smoothness assistance does not provide positive mechanical work on average. The last analysis was performed by computing the mechanical work transferred from the robot to the patient during each cycle:

29 $$\begin{array}{@{}rcl@{}} W_{patient,cycle} &=& \int^{cycle} \boldsymbol{F}_{\boldsymbol{patient}}\mathrm{d} l  \end{array} $$

where d*l* represents the infinitesimal trajectory of the patient hand, and ***F***_***patient***_ is the actual force being measured at the interface between the patient and the robot. The numerical parameters used during the therapy are provided in Table 1 (“pat. data” column).

#### Data acquisition

Data were acquired by the robot at a frequency of 250 Hz and exported in a text file with a sampling frequency of 125 Hz. The handle position and velocity were directly taken from the motor drivers with no extra filtering or signal processing.

## Results and discussion

### Validation with simulated data

#### Adaptive oscillator

**Fig. 5:**
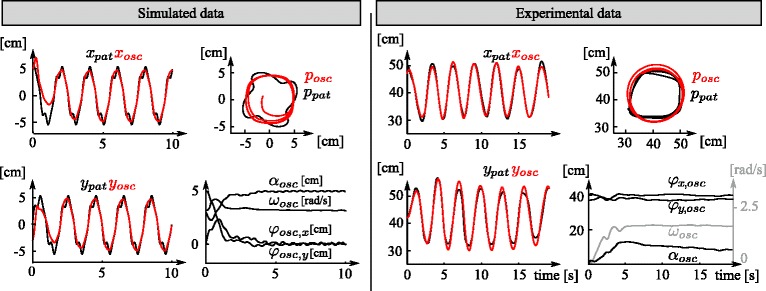
Convergence of the adaptive oscillator with simulated and experimental data. The two left graphs of both panels (simulated data and experimental data) display the oscillator input (*left*: simulated; *right*: actual position of the patient; *black line*) and the learned position by the adaptive oscillator (*red line*) as *x* and *x*_*osc*_ (*top*) and *y* and *y*_*osc*_ (*bottom*) as a function of time. The *top right* panel displays the actual movement in space (*x* and *y* axes) and the trajectory being estimated by the adaptive oscillator during 3 s. The *bottom right* panel displays the adaptive oscillator state variables as a function of time as the offsets *φ*_*x*,*osc*_, and *φ*_*y*,*osc*_ and the amplitude *α*_*osc*_ in [cm], the phase *ϕ*_*osc*_ in [rad] and the angular velocity *ω*_*osc*_ in [rad/s]. After two cycles, the oscillator synchronizes with the actual signal and gives a smooth version of the input circle (*left*: simulated; *right*: drawn by the patient)

Simulation data confirm the capacity of the adaptive oscillator to quickly synchronize to an input rhythmic movement while learning its features in state variables. Figure 5 (left) shows an illustration of the adaptive oscillator synchronizing with the simulated data with a mean amplitude of $\bar {\alpha }=5\,cm$ and mean angular velocity of $\bar {\omega } = \pi rad/s$. This simulated signal was distorted both with longitudinal (*δ*_*long*_=0.6 and $\omega _{long} = 4\bar {\omega }$) and lateral errors (*δ*_*lat*_=0.2 and $\omega _{lat}=4\bar {\omega }$). The state-variables are also displayed and reach steady-state after about two cycles (3 seconds), which corresponds to the expected settling time (see Table 1 and [56]). The oscillator output produces a smooth quasi-sinusoidal version of the distorted input and is fully synchronized after the same amount of time.

This result confirms that the state-variables *ω*_*osc*_ and *α*_*osc*_ are good candidates for estimating the real-time frequency and amplitude of quasi-harmonic movements performed by the patients. They can also be extracted in real-time during the therapy given the low computational cost of the discrete-time integration of the oscillator state equations. Interestingly, these state variables are also good candidates for assessing the initial ability of the patient to perform circular movements before receiving the therapy (see “Proposed therapy” section).

#### Validation of the real-time smoothness index

**Fig. 6:**
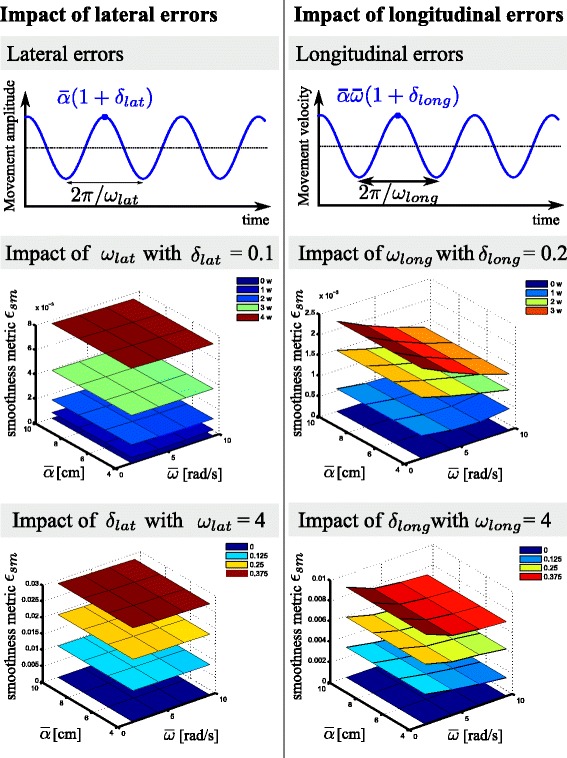
Real-time smoothness metric as a function of lateral and longitudinal errors. The *left* panels present the impact on the metric due to lateral errors (errors in amplitude), and the *right* panels show the impact due to longitudinal errors. The *two upper* panels display the simulated variation of amplitude and velocity (the product of amplitude and frequency), while the *middle* panels represent the metric variation as a function of the error frequency (fixed error amplitude), mean signal frequency, and mean signal amplitude, and the *bottom* panels present the metric variation as a function of the error amplitude (fixed error frequency), mean signal frequency, and mean signal amplitude

Figure 6 shows the impact of longitudinal and lateral errors on the metric quantifying movement smoothness. These graphs were generated by varying both the mean amplitude and angular velocity of the circles and the frequency or amplitude of the longitudinal or lateral error. Longitudinal error mostly impacts the signal temporality, while lateral errors mostly impact the signal spatial distribution.

The figure shows that the smoothness metric is insensitive to the mean movement amplitude and frequency during movements being distorted with lateral errors. This is visible in both left panels of Fig. 6, which show no variation of the metric with the mentioned parameters (i.e., flat surfaces). This capacity to predict movement smoothness independently of the movement amplitude and frequency is highly desirable. It complies with efforts made to construct a smoothness metric for discrete movements that is dimensionless and independent of the movement amplitude and duration [18, 20, 59]. With longitudinal errors, the smoothness metric is also insensitive to the average movement amplitude but displays small sensitivity to the movement frequency (right panels of Fig. 6). This is due to inherent non-linear dynamic couplings between the state variables of the adaptive oscillator.

#### Validation of the performance-based assistance

**Fig. 7:**
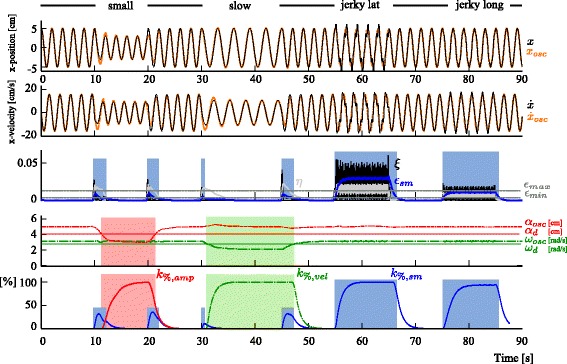
Comparison of the performance-based assistance with simulated data. The simulated data is composed of a succession of 5 periods of unperturbed sinusoidal movements and 5 periods of simulated pathological movements. The pathological movements are first small movements, followed by slow movements, jerky movements with lateral errors, and finally jerky movements with longitudinal errors. The *upper* panel shows the position signal along the *x*-axis, and the *second* panel shows the velocity along the *x*-axis. The *third* and *fourth* panels display the signals used to assess the motor performance. The *third* panel shows the signals involved in computing the smoothness metric (*η*, *ξ*, *ε*_*sm*_ and *ε*_*sm*,*min*_), and the *bottom* panel shows the adaptive oscillator state variables *ω*_*osc*_ and *α*_*osc*_ together with the corresponding minimum desired values *ω*_*d*_ and *α*_*d*_. The corresponding assistance gain increases when the performance is lower than the desired threshold, as displayed in the bottom panel. This panel shows the evolution of the three assistance gains *k*_*%*,*sm*_, *k*_*%*,*vel*_, and *k*_*%*,*ampl*_. When the corresponding error is above the threshold, a color background is added (smoothness in *blue*, velocity in *green*, and amplitude in *red*)

Figure 7 shows the evolution of the gains of the three types of assistance as a function of their corresponding errors (smoothness error, velocity error, and amplitude error; bottom panel). From top to bottom, the different panels show (i) the position signals, (ii) velocity signals, (iii) absolute error in velocity *η*, its time derivative *ξ*, and the smoothness metric *ε*_*sm*_ (the filtered version of *ξ*), and (iv) the evolution of *α*_*osc*_ and *ω*_*osc*_ with respect to the desired amplitude *α*_*d*_ and angular velocity *ω*_*d*_. The three assistance gains respond as expected. When the error of the corresponding assistance is above *ε*_*min*_, the assistance increases. If the error goes above *ε*_*max*_, the gain saturates to 1 with a settling time of about 1 s.

### Experimental validation with patient data

#### Initialization

The adaptive oscillator were used to assess the patient abilities at the beginning of the therapy. Patients were asked to execute ten circles with their paretic arm, at comfortable velocity and amplitude while receiving no assistance. Meanwhile, the adaptive oscillator learned the corresponding movement features with a small time constant (see Table 1). Figure 5 (right) shows a representative result of this adaptation in the form of the adaptive oscillator trajectory, *x*_*osc*_ and *y*_*osc*_, synchronizing to the actual position data *x* and *y*. For this particular patient and trial, the initialization finished with the oscillator state variable equal to *α*_*osc*_=9.1 cm and *ω*_*osc*_=1.71 rad/s. Consequently, setting the target movement amplitude and longitudinal velocity to 10 *%* higher during the therapy is achieved by setting *α*_*d*_=1.1*α*_*osc*_=10.01 cm and *ω*_*d*_=*ω*_*osc*_ (remember that the longitudinal velocity is the product of the amplitude *α*_*d*_ and frequency *ω*_*d*_). The patient initial average longitudinal velocity was thus equal to *v*_*osc*_=*ω*_*osc*_*α*_*osc*_=15.56 cm/s, while the target velocity used for the therapy was 10 % higher: *v*_*d*_=*α*_*d*_*ω*_*d*_=17.1 cm/s. Consequently, the patient is encouraged to perform both larger and faster movements than those executed during the initialization phase.

#### Performance-based assistances adapt to real-time motor performance

**Fig. 8:**
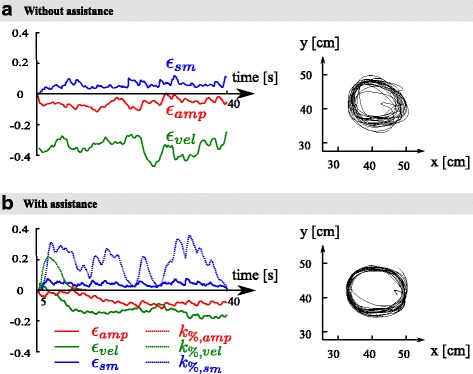
Performance with experimental data. Panels **a** and **b** show trials without and with assistance, respectively. The *left* displays the smoothness, velocity, and amplitude error and gain signals, and the *right* shows the corresponding 2D hand paths

Figure 8 illustrates the evolution of the error signals (*ε*_*sm*_, *ε*_*vel*_, *ε*_*amp*_; Eqs. (12), (13), and (14)) during a trial without assistance (upper panel) and with assistance (bottom panel). The bottom panel also shows the evolution of the level of smoothness assistance. Both tested patients showed a consistent ability to execute the circular movements at desired amplitude (*α*_*d*_) and speed (*v*_*d*_). Only a small amount of amplitude or velocity assistance was provided at the beginning of some trials, but this never lasted longer than 5 seconds. Maybe this reveals that 10 % of increase with respect to the natural velocity and amplitude was not high enough to make the movement really challenging. In contrast, the smoothness assistance was active during all trials. The assistance decreased when the smoothness metric *ε*_*sm*_ was close to 0 and increased when the smoothness metric increased.

Interestingly, the low use of the amplitude and velocity assistance by the patients could also reveal an interaction between the assistance modes. This suggests that providing smoothness assistance also had a positive impact on the steady-state movement amplitude and velocity. However, this observation should be investigated further with a larger population.

#### Performance-based assistance enhances motor performance during training

**Fig. 9:**
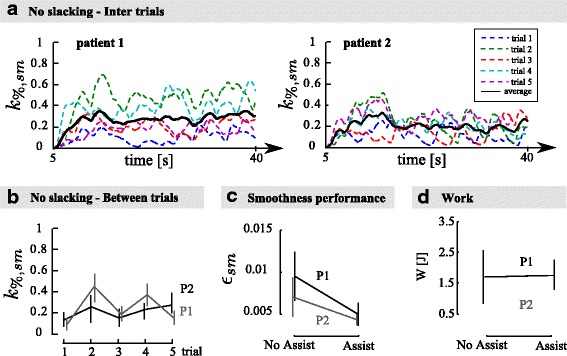
Patient data. **a** Evolution of the smoothness gain along the 5 assisted trials for both patients. The averaged evolution over the 5 trials is displayed in black. **b** Mean gain for each assisted trial for both patients. **c** Mean real-time smoothness metric during the trials with and without assistance for both patients. **d** Mean mechanical work provided by the robot to the patient during one cycle with and without assistance for both patients. Vertical lines in panels **b**, **c**, and **d** capture the patient standard deviation across trials

On the right side of Fig. 8, the hand trajectory during a trial with and without assistance is traced. This clearly shows that the hand trajectory was smoother when assistance was provided. This observation can be quantified by measuring the mean smoothness errors during the trials with and without assistance (Fig. 9c). As expected, the error was significantly higher during the trials without assistance than with assistance for both patients (one-way anova, assistance effect: patient 1, *F*_(1,8)_=9.89, *p*=0.01; patient 2, *F*_(1,8)_=5.96, *p*=0.04).

This result shows that the smoothness assistance had the expected impact on motor performances, by improving the smoothness feature of both patients movements.

#### Performance-based assistances keep the patient active

The slacking hypothesis suggests that when receiving too much assistance, a patient relies on the assistance and stops providing the maximum effort to execute the task alone [36]. This can be observed both within a trial and between trials. If patients display slacking behavior, the amount of assistance should increase over time. The evolution of the smoothness assistance gain is displayed in Fig. 9a (inter trials) and b (between trials) for both patients. Figure 9a shows the amount of assistance provided during the five assisted trials for both patients (colored dashed lines) and their average (black solid line). The figure clearly illustrates that the assistance did not increase over time during the trials, except during the initialization phase where the level of assistance reached steady-state. Figure 9b shows the mean assistance provided during each trial and confirms that the assistance was also not increasing across the 5 trials. These results show that the patient stayed equally active during the successive trials. Indeed, if they would have displayed a slacking behavior, the movement would have been degraded and the assistance gains would have increased accordingly.

Since both patients maintained the desired amplitude and velocity, the amplitude and velocity assistance did not turn on. Therefore, conclusions about a possible slacking behavior with these modes of assistance would require more experiments. Slacking is however unlikely since these assistances are based on same principles as for the smoothness assistance.

#### Smoothness assistance does not provide positive mechanical work, on average

Figure 9d illustrates the average mechanical work delivered by the patients to the robot during the execution of one movement cycle with assistance and without assistance (see Eq. (29)). This mechanical work was positive on average, revealing that the robot was actually dissipating energy in both the transparent mode (no assistance) and when the assistance was switched on. This is due to the residual robot friction *b*, which was not entirely hidden by the robot force controller. Patient 1 delivered the same amount of energy to execute the circles with and without assistance (one-way ANOVA; assistance effect: *F*_(1,8)_=0.13, *p*=0.73). Patient 2 delivered a little bit more energy with assistance than without, although this did not reach significance (*F*_(1,8)_=3.54, *p*=0.09).

This result indicates that our smoothness assistance obeys passivity-like properties, as it does not provide mechanical energy to the patient on average. Passivity has recently emerged as a highly desirable feature for rehabilitation robots since it does not compromise the stability of the patient-robot interaction once they are connected to each other [63–66].

### Perspectives

The data that were reported in the present paper were acquired for the purpose of validating our approach with two representative pilot patients. Consequently, this opens several perspectives for future research. The first is to validate our assistance principle with a larger stroke population, in order to confirm the reported preliminary results. The second is to explore the short- and long-term effects of the suggested unilateral rhythmic movement training on the motor performances of stroke patients. Moreover, possible improvements should also be quantified on other types of movements, like discrete reaching, although the litterature established that there is few to no learning transfer from rhythmic to discrete movements in healthy subjects [13, 14]. Interestingly, our previous study suggested that stroke patients tend to perform their rhythmic movements kinematically close to discrete movements of healthy subjects [24]. Therefore, investigating possible transfer of learning between rhythmic and discrete movements in a stroke population is of high interest, in particular regarding movement features like smoothness. In sum, our future investigations will aim at establishing which movement features could be improved, both in rhythmic and discrete primitives, after a unimanual rhythmic movement training. The assistance presented in this paper is proposed as a candidate for implementing such a robot-assisted rhythmic movement training.

In parallel, a progression in the exercises could also be proposed, owing to the fact that our recent findings also revealed smaller impairments of rhythmic movements in a typical stroke population [24]. Discrete elements could be combined with rhythmic movements, supporting the execution of movements with a higher degree of impairment by those that are performed more stably. The combination of rhythmic and discrete movements, like those performed by [67] for single-joint movements or [8] for two-joint movements, are viable candidates for this type of training. Our performance-based assistance could assist the rhythmic component of such movements, although it would have to deal with the reported conflicts between both neural controllers that are being trained. For instance, [8] revealed that the discrete component of a combined rhythmic+discrete movement is systematically triggered during a limited phase window of the rhythmic one.

Finally, the presented performance-based assistance could be extended to more functional rhythmic movements such as wiping a table, brushing teeth or even walking [54]. Indeed, the assistance principle is applicable to any rhythmic movement performed with an end-effector robot, but also with an exoskeleton.

## Conclusion

Rhythmic and discrete movements are considered to be two different motor primitives, and both are affected after stroke. Therefore, both movements should be trained during post-stroke therapies. Currently, mainly functional discrete movements are used during post-stroke therapies, and similarly, the majority of assistance methods being developed for rehabilitation robots target only discrete movement training. To bridge this gap, our study presented a new performance-based robotic assistance training for rhythmic movements that should ideally complement similar discrete-specific therapies in order to recover the most complete motor repertoire.

The proposed assistance method relies on the repetitive nature of rhythmic movements to independently assist three movement features, i.e. smoothness, velocity, and amplitude, without constraining the participant to follow a predefined trajectory. Three assistance forces are combined in order to enhance these features. The forces are modulated as a function of corresponding real-time metrics and therefore assist the patient as needed to prevent slacking. Our approach ideally combines several blocks: an adaptive oscillator that extracts the real-time movement features while providing the movement main harmonic, three estimators that compute the real-time movement features, and three adaptive blocks that compute the feature-based assistance forces. Notably, these blocks consist of a few dynamic equations and can thus be implemented in a discrete-time version with a limited amount of code and reasonable computational cost.

These different blocks were tested using both simulated and actual experimental data. The simulation data mainly served to validate the independent and decoupled computing of the different movement features using signals with controlled distortions. In particular, the proposed real-time smoothness metric proved to quantify movement smoothness while being independent of the mean movement amplitude and frequency. Patient data revealed the method efficiency in assisting patients to produce smoother movements while keeping them active in the task. In particular, we showed that the robot did not provide mechanical energy to the patient on average such that our strategy displayed passivity-like properties.
